# Enhancing the Catalytic Performance of β-Mannanase via Polyvinyl Alcohol Immobilization and Genipin Modification

**DOI:** 10.3390/molecules30234567

**Published:** 2025-11-27

**Authors:** Nazli Ece Varan Faki, Ali Toprak, Guzide Yucebilgic, Dilek Alagöz, Deniz Yildirim, Roberto Fernandez-Lafuente

**Affiliations:** 1 Chemistry Department, Sciences & Letters Faculty, Cukurova University, Adana 01330, Turkey; evaran@cu.edu.tr (N.E.V.F.); gybilgic@cu.edu.tr (G.Y.); dyildirim@cu.edu.tr (D.Y.); 2 Acigol Vocational School, Nevsehir Haci Bektas Veli University, Nevsehir 50140, Turkey; alitoprak@nevsehir.edu.tr; 3 Imamoglu Vocational School, Cukurova University, Adana 01330, Turkey; 4 Chemical Engineering Department, Engineering Faculty, Cukurova University, Adana 01330, Turkey; 5 Departamento de Biocatalisis, ICP-CSIC, 28049 Madrid, Spain

**Keywords:** anticancer effect, enzyme stability, genipin, immobilization, mannanase, MOS

## Abstract

This study reports the immobilization of β-mannanase from *Aspergillus niger*—either unmodified or genipin-modified—within polyvinyl alcohol hydrogels (PVA@mannanase and PVA@mannanase-Gen) for the enhanced production of mannooligosaccharides (MOSs). All enzyme preparations showed an optimal pH of 5.0, while immobilization shifted the optimal temperature from 40 °C for the free enzyme to 55 °C for the immobilized forms. Genipin modification notably improved stability, increasing the half-life from 25.3 h (free enzyme) to 429.2 h in PVA@mannanase-Gen, and raised catalytic efficiency by approximately 2.3-fold. Both immobilized preparations retained over 75% of their activity after five reuse cycles at pH 5 and 55 °C. Using PVA@mannanase-Gen under these optimized conditions, MOSs were effectively produced, with mannotetraose as the predominant product. To explore their potential applications, the MOSs generated from locust bean gum were evaluated for effects on MCF-7 and HCT-116 cancer cell lines, resulting in moderate growth inhibition (~24–25% at 0.4 mM after 24 h). Together, these findings demonstrate that the immobilization of the genipin-modified enzyme not only enhances β-mannanase stability and performance but also supports the efficient production of MOSs with promising antitumoral activity.

## 1. Introduction

Endo-1,4-mannanases (E.C. 3.2.1.78) are enzymes that catalyze the hydrolysis of the (1,4)-D-mannosidic linkages found in mannans and related heteropolysaccharides, such as galactomannans and glucomannans [1,2]. This enzymatic activity results in the production of smaller mannooligosaccharides, which are composed of 3–10 mannose residues. These enzymes play a significant role in the degradation of plant materials, making them important in various industrial applications, including food processing, animal feed, and biofuel production [3,4]. Their ability to break down complex carbohydrates can enhance the digestibility of feed and improve the yield of fermentable sugars in bioconversion processes [5,6]. However, the use of free enzymes for industrial applications can be limited by several factors, including instability at elevated temperatures, reduced activity at high substrate concentrations, susceptibility to inhibition by chemicals used in production, etc. [5]. To enhance their performance, modifications are often necessary prior to application. A widely adopted and practical solution to these issues is the immobilization of enzymes. An adequate use of this technique not only stabilizes enzymes under harsh conditions [7,8,9,10,11,12,13] but also allows their easier recovery and reuse [14], significantly improving their efficiency and effectiveness in various industrial processes [15,16,17,18]. Enzyme immobilization may be used for a final tuning of the enzyme features (selectivity, specificity, resistance to inhibitors) [19,20,21] or may be coupled to enzyme purification [22]. That way, enzyme immobilization is far more than a mere tool to recover and reuse the enzymes, but it is a last opportunity of tuning the enzyme features [23]. In the case of β-mannanase, these advantages are especially valuable because its industrial applications—such as biomass hydrolysis and mannooligosaccharide production—often require elevated temperatures, prolonged operation times, and exposure to viscous, inhibitor-rich substrate matrices. Consequently, various immobilization strategies—employing supports such as polymeric matrices, inorganic materials, biocompatible gels, and a wide range of nanomaterials—have been explored to enhance the robustness and efficiency of β-mannanases from different sources. Al Shimaa Gamal Shalaby et al. [24] immobilized mannanase from *Penicillium chrysogenum* in calcium alginate beads and reported that both the free and immobilized mannanase preparations successfully hydrolyzed locust bean gum to valuable mannooligosaccharides. Panwar et al. [25] prepared crosslinked enzyme aggregates (CLEAs) of endo-β-1,4-mannanase and demonstrated that the immobilized mannanase showed better pH and thermal stabilities than those of the free counterpart. Behera et al. [26] utilized the same immobilization strategy to immobilize crude β-mannanase and found that the prepared CLEAs were active over a broader pH range than the free enzyme. Sadaqat et al. [27] used glutaraldehyde crosslinked chitosan beads to immobilize β-mannanase and determined that the immobilized mannanase had a higher optimal temperature than the free enzyme. Dhiman et al. [28] immobilized partially purified β-mannanase on the modified matrix of sodium alginate-grafted β-cyclodextrin and showed that the immobilization increased the thermostability of the β-mannanase. Dikbaş et al. [29] immobilized mannanase from *Bacillus invictae* onto zinc oxide nanoparticles and determined that the immobilized mannanase preparation had positive effects on the germination parameters of tomatoes.

Polyvinyl alcohol (PVA) is a widely used polymer for enzyme and cell immobilization due to its affordability, ease of availability, hydrophilic nature, and the presence of surface hydroxyl groups [18,30,31,32,33,34]. These properties make PVA an excellent matrix for various biocatalysts [35,36]. Additionally, functionalizing PVA or combining it with other materials can further enhance the catalytic properties of the immobilized enzymes, improving their operational stability and overall performance in industrial applications. Such modifications can lead to increased enzyme activity, higher resistance to harsh conditions, and prolonged reusability, making PVA a versatile choice for enzyme immobilization [37,38].

Genipin is a naturally occurring crosslinking agent that facilitates the formation of three-dimensional networks by crosslinking molecules [39,40]. This crosslinking network formation enhances the stability and structural integrity of the materials used in various applications. The use of spacer arms plays a critical role in modulating the catalytic performance and stability of immobilized enzymes [41]. Flexible or multifunctional linkers such as (3-Aminopropyl)triethoxysilane (APTES), genipin, glutaraldehyde, and polyethyleneimine (PEI) reduce steric hindrance, improve the accessibility of active sites, and create a microenvironment that supports more native-like enzyme conformations [42]. Genipin is a safer and more biocompatible alternative to conventional crosslinking systems such as PEI, APTES, and glutaraldehyde. Unlike glutaraldehyde, genipin provides stable covalent networks with a significantly lower toxicity, making it more suitable for cell-compatible scaffolds and in vivo applications [27,43]. Although PEI offers dense amine functionality, it is highly cytotoxic and prone to non-specific interactions [44]. APTES is effective for silanizing inorganic surfaces; however, its hydrolytic instability and lack of true crosslinking capacity limit its usefulness in forming robust 3D networks [45]. Overall, while genipin crosslinking is slower and may result in blue pigmentation, its biocompatibility, controlled reactivity, and ability to stabilize natural polymers make it a superior choice for tissue engineering, hydrogel formation, and bioactive scaffold design [46,47].

Mannooligosaccharides (MOSs) may be produced through the enzymatic, alkaline, or acidic hydrolysis of cell wall mannans derived from different sources such as yeast (*Saccharomyces cerevisiae*) or vegetable galactomannans [48,49,50]. MOSs, which consist of linear chains of mannose, can be further classified into α- and β-MOSs. β-MOSs are primarily used as prebiotics in the poultry industry [50]. Recent studies indicate that MOSs may serve as an important functional food ingredient and hold potential applications as antioxidants and anti-cancer agents [51]. MOSs are reported to have prebiotic properties due to their resistance to digestion in the upper gastrointestinal tract [52]. Additionally, MOSs are noted for their potential antineoplastic (anti-cancer) properties, which may help to inhibit the growth of cancer cells [53]. Importantly, MOSs have been shown to exert these effects without causing cytotoxicity to normal, healthy cells, making them a promising natural compound for both gut health and cancer prevention [51].

MCF-7 cells (a human breast cancer cell line) are one of the most widely used cell lines in cancer research worldwide [54]. MCF-7 cells serve as a model for evaluating chemotherapeutic agents, targeted therapies, and novel anti-cancer drugs. Their responses to various agents can be measured through changes in cell proliferation, apoptosis, and migration, making them suitable for screening antitumoral drug candidates [55]. The HCT-116 cell line is a human colorectal carcinoma cell line also used in cancer research. It was derived from a primary colon adenocarcinoma tumor and has been extensively studied for its relevance in understanding colon cancer biology, drug development, and cellular mechanisms [56]. Pason et al. [57] showed that MOSs from coconut meal inhibit the apoptosis of HCT116 cells.

Although several immobilization strategies have been applied to β-mannanase, the existing approaches present several limitations. Many rely on harsh or potentially toxic crosslinkers such as glutaraldehyde, which can restrict biocompatibility and influence downstream applications, particularly when the target products—such as MOSs—are intended for food, nutraceutical, or biomedical uses. Moreover, while structural stabilization has been achieved, fewer studies have focused on immobilization methods that simultaneously enhance enzyme stability, catalytic efficiency, and product selectivity. The influence of immobilization on MOS composition, especially the targeted production of higher-value oligosaccharides, also remains insufficiently explored. In this context, genipin-based immobilization offers a promising alternative, combining effective crosslinking capacity with low toxicity, improved biocompatibility, and the potential to modulate enzyme microenvironments more gently than conventional agents. By integrating genipin modification with PVA hydrogel immobilization, the present study aims to address key gaps in the field—namely, achieving enhanced stability at elevated temperatures, improving catalytic efficiency, and employing a more sustainable and application-friendly immobilization strategy for the production of MOSs.

In this study, mannanase from *Aspergillus niger* was encapsulated in PVA (PVA@mannanase), and the obtained PVA@mannanase preparation was subjected to modification with genipin (PVA@mannanase/Gen). β-mannanase from *A. niger* was chosen due to its commercial availability, known high activity, and previous successful applications in MOS production [58,59]. All mannanase samples were characterized in terms of optimal pH, temperature, thermal stability, and kinetic parameters. Locust bean gum (LBG) hydrolysis reaction courses catalyzed by PVA@mannanase/Gen were monitored by determining the amount of reducing sugar, and the formed MOSs were determined using high-performance liquid chromatography. The produced MOSs were tested as inhibitors of the growth of cancer cells of MCF-7 and HCT-116 cell lines.

## 2. Results and Discussion

Table 1 shows the immobilization yields (%) and expressed activity (%) of PVA@mannanase and PVA@mannanase-Gen for different protein loadings. The immobilization yields were 100% for all assayed protein loadings in the range of 0.125–0.5 mg. The modification of the enzyme with genipin (2 g/L) produced an improvement of the immobilized enzyme activity at the three assayed loadings. The study of the activity of the free enzyme modified by genipin showed that this modification caused an increase in the activity of the enzyme in the range of 110–125%. This could be a consequence of the promotion of favorable changes in the enzyme structure caused by the modification, or of a stabilization of the enzyme, which permitted the modified enzyme to show a higher activity under the relatively harsh conditions utilized in the enzyme activity determination. This higher activity after genipin modification was transferred to an improved enzyme activity after the immobilization of the modified enzyme, which, when using 0.25 mg/mL, was even higher than the activity of the free and unmodified enzyme. Curiously, the highest expressed activities were determined at the intermedium enzyme loading (0.25 mg/mL) for the modified and unmodified immobilized enzyme preparations, reaching 86.6% and 119.2% of the offered enzyme activity for PVA@mannanase and PVA@mannanase-Gen, respectively, when the initial protein loading was 0.25 mg/mL. The higher expressed activity when the load increased from 0.125 to 0.25 mg/mL was not expected (as the substrate diffusional matter should produce an enzyme decrease in activity when the volumetric activity increases) [60,61,62], and may be due to diverse facts. One possibility is that the free enzyme may be more stable at higher concentration by forming enzyme aggregates. These enzyme aggregates may be more stable than the individual enzyme molecules, and they should be larger. After immobilization, if the enzyme aggregates are immobilized together with individual enzyme molecules, it is possible that the smaller individual enzyme molecules can be washed from the beads, while the larger enzyme aggregates remain immobilized. These aggregates can be more abundant or larger using higher enzyme concentrations, while aggregates that are too large can suffer from additional substrate diffusion limitations. Another possibility is the fact that the determination of the enzyme activity is based on the increase in reducing sugars, not the degradation of new mannan molecules. It is possible that the smaller fragments of the mannans may become better substrates than the intact polymer, and the enzyme molecules immobilized inside the particles can attack these fragments, producing a higher amount of reducing groups even with the modification of a lower amount of the mannan molecules [63]. The use of a further increased amount of enzyme can lead to an increase in the substrate diffusional limitations and produce a decrease in enzyme activity, even in the hypothetical case of a better hydrolysis of the smaller fragment of the polymer substrate. If this was the case, the use of the immobilized enzyme should produce a higher number of small MOSs at a lower initial mannan degradation. This possibility is currently under study in our research group.

**Figure 1:**
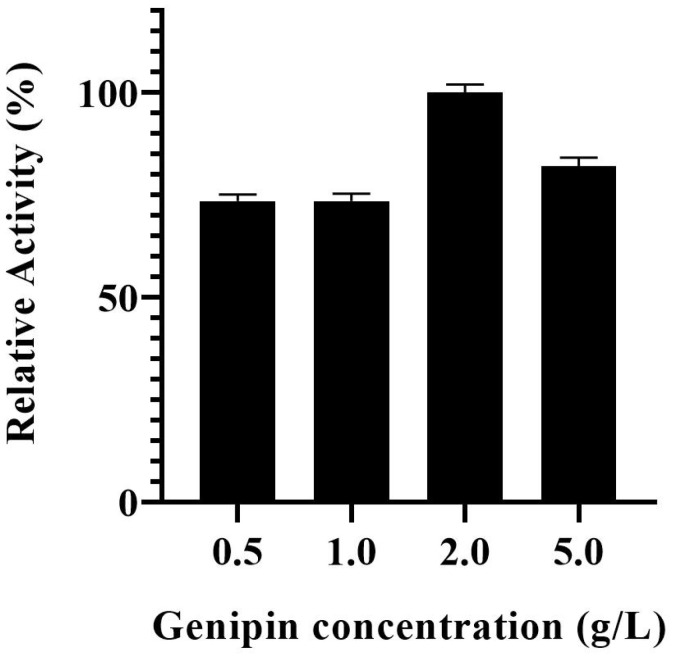
Effect of genipin concentration on the activity of PVA@mannanase-Gen. All experiments have been repeated in triplicate. Experiments were performed as described in the Methods section.

The effect of genipin concentration used in the biocatalyst modification on the activity of PVA@mannanase-Gen was studied over a concentration range of 0.5–5.0 g/L. As illustrated in Figure 1, the activity of PVA@mannanase-Gen remained unaffected when the genipin concentration was increased from 0.5 to 1.0 g/L. However, when the genipin concentration was raised to 2.0 g/L, the highest activity was observed. Further increases in the genipin concentration led to a decrease in the enzyme activity, very likely by an excessive modification of the enzyme surface. According to these results, the selected genipin concentration used for the preparation of PVA@mannanase-Gen was 2 g/L.

**Figure 2:**
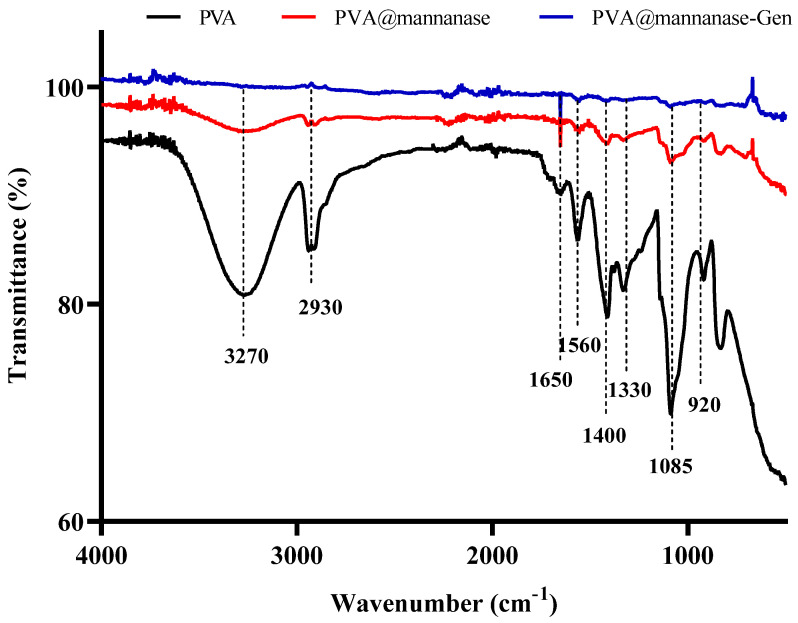
FTIR spectra of PVA, PVA@mannanase, and PVA@mannanase-Gen at 500–4000 cm^−1^.

FTIR spectra of PVA, PVA@mannanase, and PVA@mannanase-Gen are shown in Figure 2. The band around 3200–3600 cm^−1^ indicates the presence of hydroxyl groups from the PVA molecule. The peak at 2800–3000 cm^−1^ is attributed to the C-H stretching vibration from the PVA backbone. The peak at 1050–1150 cm^−1^ corresponds to the C-O stretching vibration, associated with the alcohol (-OH) groups in PVA [64]. After the immobilization of mannanase, the intensity of band around 3200–3600 cm^−1^ decreased. This may be due to the formation of hydrogen bonds between the free hydroxyl groups of PVA molecules and enzyme molecules. The peak produced using PVA@mannanase or PVA@mannanase-Gen around 1650 cm^−1^ is attributed to the amide I bond, indicating the presence of mannanase in the composite.

**Figure 3:**
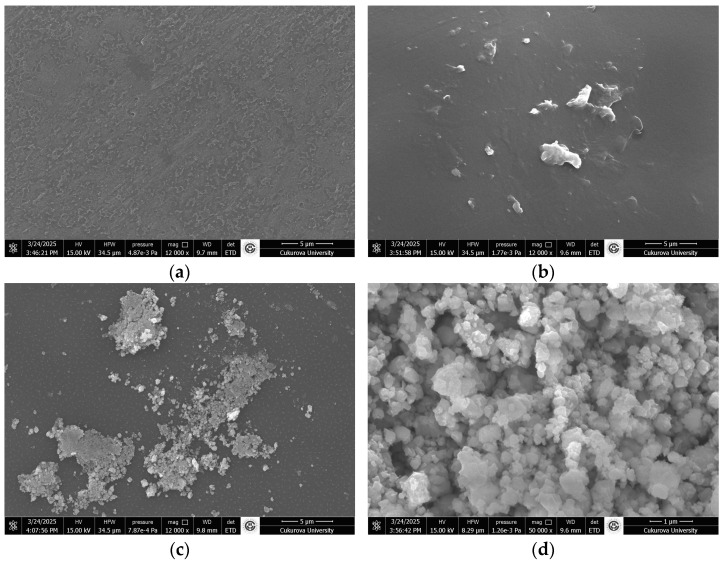

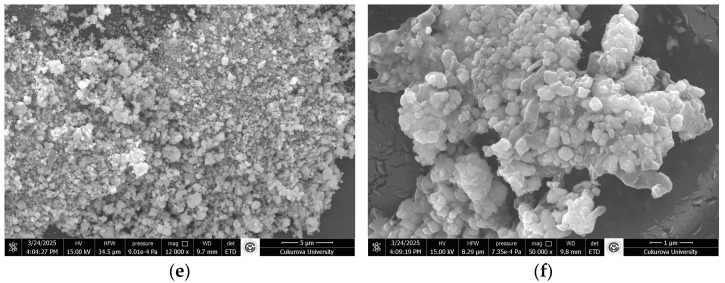
SEM images of PVA (**a**,**b**), PVA@mannanase (**c**,**d**), and PVA@mannanase-Gen (**e**,**f**). Other specifications can be found in the Methods section. The experiments were performed as described in the Methods section.

SEM images of PVA, PVA@mannanase, and PVA@mannanase-Gen may be found in Figure 3. The PVA surface was smooth, dense, and non-porous (Figure 3a,b), while PVA@mannanase and PVA@mannanase-Gen show a porous structure due to the interaction between the enzyme and the PVA (Figure 3c–f). This increase in porosity may cause the substrate molecule to be more accessible to the enzyme.

**Figure 4:**
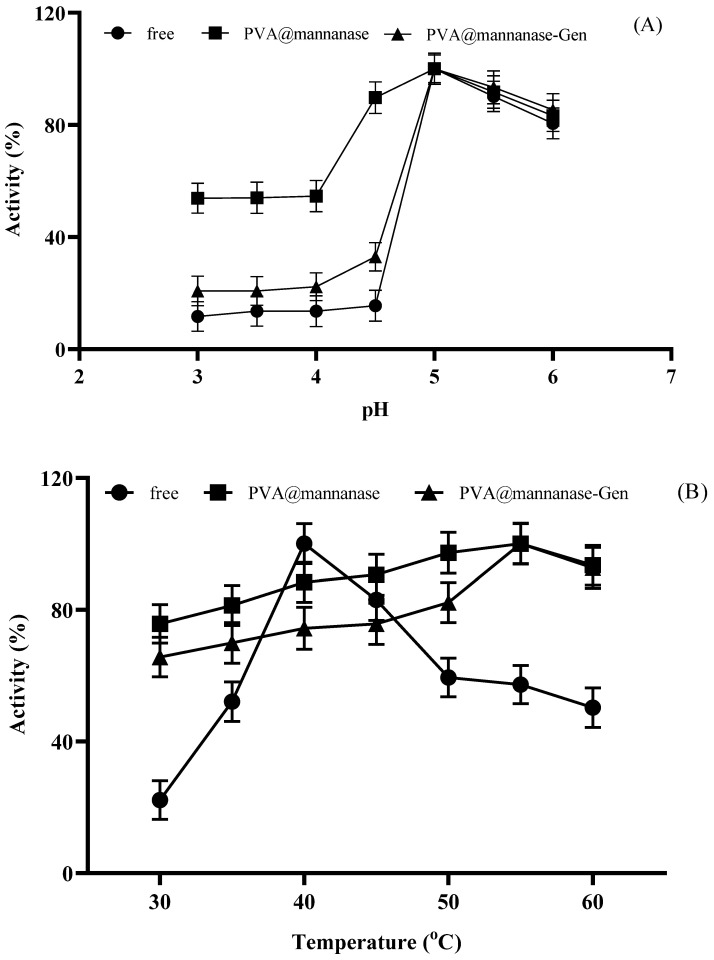

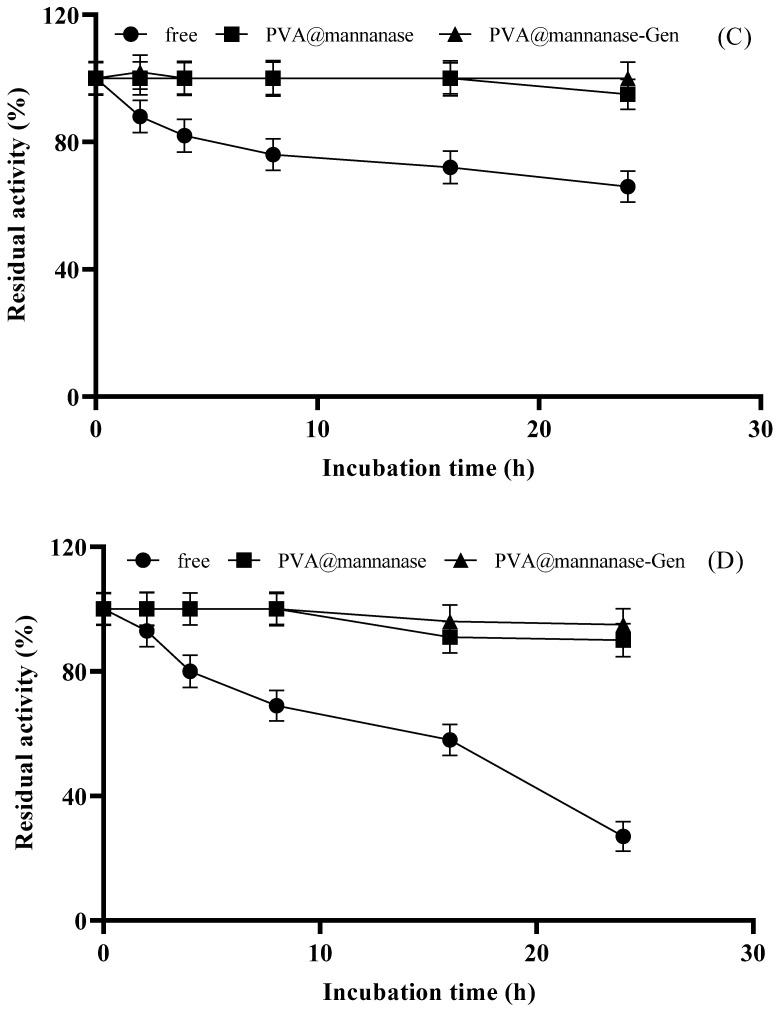
Effect of pH on the activity of free mannanase, PVA@mannanase, and PVA@mannanase-Gen at 40 °C and 0.25% (*w*/*v*) LBG concentration (**A**). Effect of temperature on the activity of free mannanase, PVA@mannanase, and PVA@mannanase-Gen at pH 5.0 and 0.25% (*w*/*v*) LBG concentration (**B**). Thermal stability of free mannanase, PVA@mannanase, and PVA@mannanase-Gen at 40 °C (**C**) and 55 °C (**D**) at pH 5.0. All experiments have been repeated in triplicate. Other specifications may be found in the Methods section.

Next, the effect of the pH on the activity of free and immobilized β-mannanase preparations was analyzed. As illustrated in Figure 4A, the highest mannanase activity was recorded at pH 5.0 for all preparations. PVA@mannanase retained much more relative activity at a pH under 5 than PVA@mannanase-Gen or the free enzyme. PVA@mannanase-Gen retained more activity than the free enzyme at these acidic pH values. However, when the pH values were higher than 5, the relative activities of all preparations became very similar. It should be considered that PVA@mannanase-Gen was already significantly more active than the free enzyme, so the specific activity of the enzyme in this biocatalyst was much better than that of the free enzyme in all the studied pH ranges.

As illustrated in Figure 4B, the highest activity of free β-mannanase at pH 5.0 was determined at 40 °C, whereas the highest activities of PVA@mannanase and PVA@mannanase-Gen were both at 55 °C. Notably, the activities of PVA@mannanase and PVA@mannanase-Gen were less affected by the temperature change compared to the free enzyme in the temperature range of 30–60 °C, highlighting the protective benefits of the immobilization process for the mannanase activity. The optimum pH for both forms of the Man/Cel5B enzyme was found to be pH 5.5. The optimum pH and temperature of both free β-mannanase and its immobilized form on the zinc oxide nanoparticle were 6.0 and 40 °C, respectively [29]. Blibech et al. [65] reported that the optimal pH of β-mannanase produced from five soil-derived *Bacillus* strains was 5.0, and the optimal temperature was in the range of 50–70 °C. Zhao et al. [66] found that the optimal pH and temperature of β-mannanase purified from *Lactobacillus casei* HDS-01 were 5.0 and 40 °C, respectively.

Next, the thermal stability of free mannanase, PVA@mannanase, and PVA@mannanase-Gen was examined at 40 °C and 55 °C for 24 h (Figure 4C,D). The results indicated that PVA@mannanase and PVA@mannanase-Gen retained 95% and 100% of their initial activity after 24 h of incubation at 40 °C. However, the retained activity for the free mannanase was 66% of its initial activity under the same conditions. At 55 °C, the activity of free mannanase decreased significantly, and the retained activity was approximately 27% after 24 h of incubation. However, PVA@mannanase and PVA@mannanase-Gen remained almost fully active after this incubation (90% and 95%, respectively). These findings clearly demonstrate that the immobilized β-mannanase exhibits enhanced stability. This also may explain the significant increase in the optimal temperature at pH 5.0 detected for these immobilized biocatalysts. Moreover, the modification of PVA@mannanase with genipin resulted in a new increase in thermal stability, perhaps by achieving an inter-molecular crosslinking. Shalaby et al. [24] immobilized partially purified mannanase from *Penicillium chrysogenum* in calcium alginate beads and reported that the immobilized mannanase was about 2-fold more thermostable than the free mannanase at 70 °C. Anderson et al. [67] showed that the retained activities of both the free and immobilized mannanase were similar after 10 h incubation at 60 °C (approximately 75%).

The kinetic parameters for the free and immobilized β-mannanase preparations towards LBG are presented in Table 2. The apparent *K*_m_ values for the free enzyme, PVA@mannanase, and PVA@mannanase-Gen were found to be 25.8 ± 0.04, 69.6 ± 0.05, and 14.0 ± 0.03 mg mL^−1^, respectively. The corresponding *V*_max_ values were 55.0 ± 0.07, 34.4 ± 0.08, and 68.9 ± 0.06 U/mg protein. The k_cat_ values were calculated to be 44.0, 27.5, and 55.1 min^−1^ for the free enzyme, PVA@mannanase, and PVA@mannanase-Gen, respectively. The corresponding k_cat_/*K*_m_ values were 1.7, 0.4, and 3.9 s^−1^ mg^−1^ mL. According to these results, the catalytic efficiency values were calculated to be 0.24 and 2.30 comparing the free enzyme and PVA@mannanase or PVA@mannanase-Gen, respectively. PVA@mannanase shows a worsening of both the *K*_m_ (more significant) and k_cat_ compared to the free enzyme. However, PVA@mannanase-Gen shows a significant increase in efficiency compared to the free enzyme or the unmodified immobilized enzyme, because both *K*_m_ (more intensely) and k_cat_ are improved after genipin modification. As the unmodified immobilized enzyme did not improve the *K*_m_, the positive partition of the substrate can be discarded as the main cause for the improved *K*_m_ of the genipin-modified enzyme. The enhanced catalytic efficiency of PVA@mannanase-Gen arises from genipin-induced conformational stabilization and an improved microenvironment within the PVA matrix. Genipin modification seems to induce a positive conformational change in the enzyme structure, lowering the *K*_m_ and increasing k_cat_, resulting in an approximately 9.6-fold increase in catalytic efficiency compared to the unmodified enzyme. Anderson et al. [67] covalently immobilized endo-1,4-β-mannanase from *Aspergillus niger* on glutaraldehyde-activated chitosan nanoparticles, and the *K*_m_ and *V*_max_ values were 8.44 mg/mL and 55.36 U/mg protein, respectively. These values corresponded to 7.74 mg/mL and 12.10 U/mg protein for its immobilized counterpart. Panwar et al. [25] prepared the crosslinked aggregated (MB-C) and chitosan magnetic nanocomposites of MB-C (MB-Mag-C), forms of recombinant endo-β-1,4-mannanase. The *K*_m_ and *V*_max_ values of MB-C were found to be 8.1 mg/mL and 135 U/mg protein, respectively. These values for MB-Mag-C were correspondingly 6.7 mg/mL and 67.1 U/mg protein. The *K*_m_ values of free and immobilized NaAlg-β-CD-β-mannanase were determined to be 25 and 36 mg/mL. The corresponding *V*_max_ values were 2500 and 2252.25 U/mg protein [28]. The *K*_m_ values were found to be 10.2 and 19.4 mg/mL for the free β-mannanase and Man-CaAlg, respectively. The *V*_max_ values were correspondingly 3.24 and 6.17 U/mg protein [68]. The *K*_m_ values of the free and immobilized Man/Cel5B were found to be 10.7 mg/mL and 4.8 mg/mL, respectively. Furthermore, the *V*_max_ values of free and chitosan-immobilized Man/Cel5B were found to be 106 and 2.56 U/mg protein [27]. Compared to the other immobilized β-mannanases given in Table 2, PVA@mannanase-Gen exhibits a superior catalytic efficiency, reflected in its lower *K*_m_ and higher *V*_max_. Unlike other immobilization methods, this approach minimizes steric hindrance and denaturation, enhancing both substrate binding and turnover. Together, these factors enable PVA@mannanase-Gen to outperform previously reported immobilized β-mannanases.

**Figure 5:**
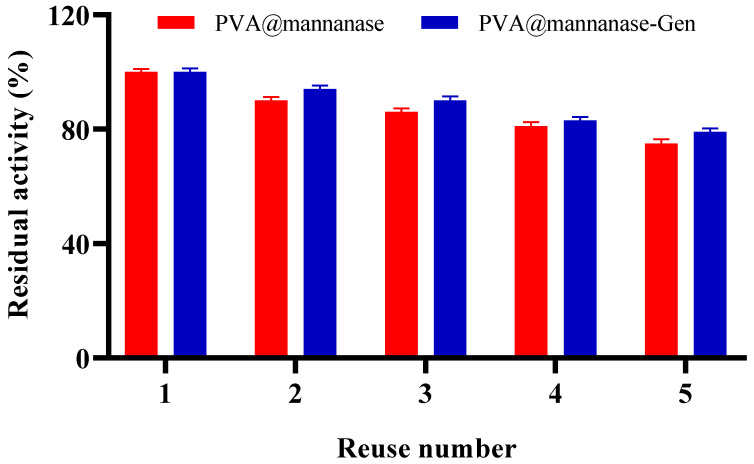
Reuse stability of PVA@mannanase and PVA@mannanase-Gen at pH 5.0 and 55 °C. All experiments have been repeated in triplicate. Other specifications may be found in the Methods section.

Figure 5 shows the operational stability of PVA@mannanase and PVA@mannanase-Gen for five consecutive reuses. The residual activity of both PVA@mannanase and PVA@mannanase-Gen slightly decreased with an increasing reuse number. After five reuses, the residual activities were 75% and 79% of the initial activity of PVA@mannanase and PVA@mannanase-Gen, respectively. This decrease in biocatalyst activity may be due to enzyme inactivation, the loss of some biocatalyst mass after each reuse in the filter, enzyme release from the biocatalyst, or the accumulation in the beads of some substances that can act as enzyme inhibitors. In the case of PVA-based hydrogels, an additional factor may be the partial clogging of pores by the large LBG substrate and intermediate hydrolysis products, which can create diffusion barriers and progressively restrict substrate access to the immobilized enzyme during repeated cycles. Chen et al. [60] reported that CaAlg maintained 70.34% of its initial activity after eight reuses. MB-C and MB-Mag-C were used to hydrolyze LBG for 12 cycles, and MB-C and MB-Mag-C retained 31.08% and 8.6% of their initial activities at the end of 12 cycles [25]. Dhiman et al. demonstrated that 70% of the residual activity was retained after 15 repeated cycles for β-mannanase immobilized on the modified matrix of sodium alginate-grafted β-cyclodextrin [28]. Sadaqat et al. [27] reported that β-mannanase immobilized on glutaraldehyde crosslinked chitosan beads remained at 70% of its initial activity after 15 uses. Compared with the examples in the literature, PVA@mannanase and PVA@mannanase-Gen have a good reusability.

**Figure 6:**
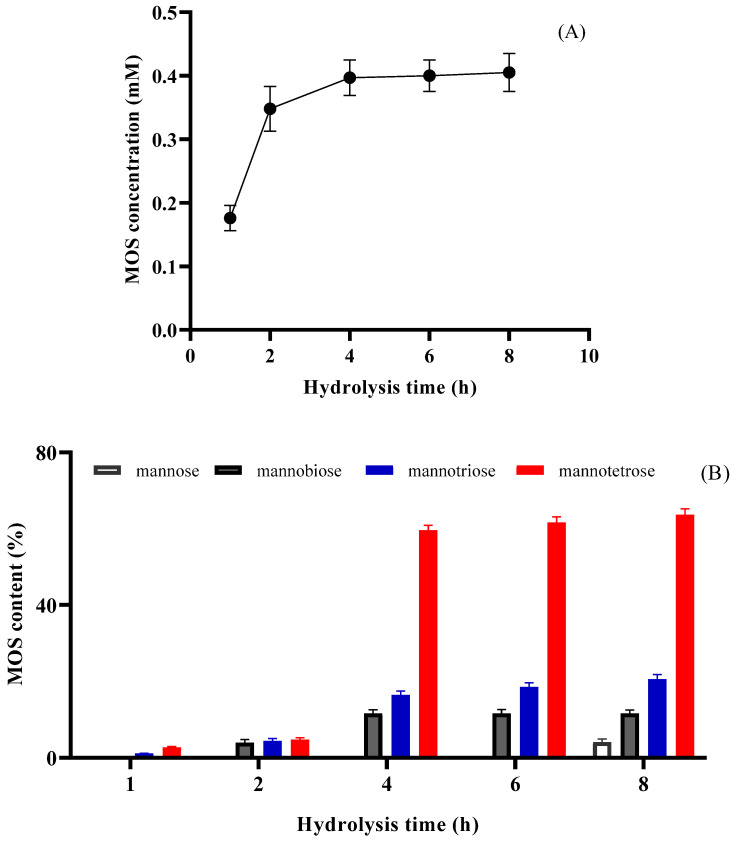
The time-course hydrolysis of LGB (0.25%, *w*/*v*) catalyzed by PVA@mannanase-Gen at pH 5.0 and 55 °C. The amount of formed MOSs (**A**) and MOS products (**B**). All experiments have been repeated in triplicate.

The time-course hydrolysis of LGB catalyzed by PVA@mannanase-Gen is shown in Figure 6A. The total amount of the MOS concentration was calculated to be 0.176 ± 0.03 mM after 1 h of hydrolysis time and linearly increased to 0.348 ± 0.05 mM at the end of 2 h of hydrolysis time. The total amount of MOSs was determined to be 0.40 mM after 6 h of reaction time, and an increase in reaction time (8 h) did not change the total MOS concentration. The MOS productivity of PVA@mannanase-Gen was calculated to be 0.05 mM/h. The products of the PVA@mannanase-Gen hydrolysis of LBG at different reaction times were also analyzed using the HPLC-RID technique. The retention times of mannose, mannobiose, mannotriose, and mannotetraose were 8.73, 9.73, 10.77, and 12.19 min. After 8 h of reaction time, the main formed MOS was mannotetraose. The yield of mannotetraose was approximately 63.7% of the total MOS amount. The yields of mannose, mannobiose, and mannotriose corresponded to 4.1%, 11.6%, and 20.6% of the total MOS amount, respectively (Figure 6B). The concentrations of mannose, mannobiose, mannotriose, and mannotetraose were approximately 0.016, 0.046, 0.08, and 0.25 mM, respectively. Chauhan et al. [69] showed that the major detected products of LBG hydrolysis catalyzed by free β-mannanase from *Bacillus nealsonii* PN-11 were mannobiose (11%), mannotriose (23%), mannotetrose (20%), and mannopentose (18%). According to the study by Sachslehner et al. [70], the mannanase from *Sclerotium rolfsii* generated mannooligosaccharides, including mannobiose, mannotriose, and mannotetrose. Rana et al. [71] reported that the yields of mannobiose (M2), mannotriose (M3), mannotetrose (M4), mannopentose (M5), mannohexose (M6), and mannoheptose (M7) were 28.9%, 23.3%, 10.6%, 15.9%, 13.2%, and 8.2%, respectively, for the hydrolysis of copra meal catalyzed by mannanase from *Bacillus subtilis* after 6 h of reaction time at pH 7.5 and 50 °C. Erkan et al. [72] calculated the amounts of M2, M3, M4, M5, and M6 as 1.86, 2.02, 0.70, 0.44, and 1.49 mM, respectively, from coffee extract after 90 min of reaction time at 40 °C using a recombinant *Aspergillus sojae* β-mannanase.

**Figure 7:**
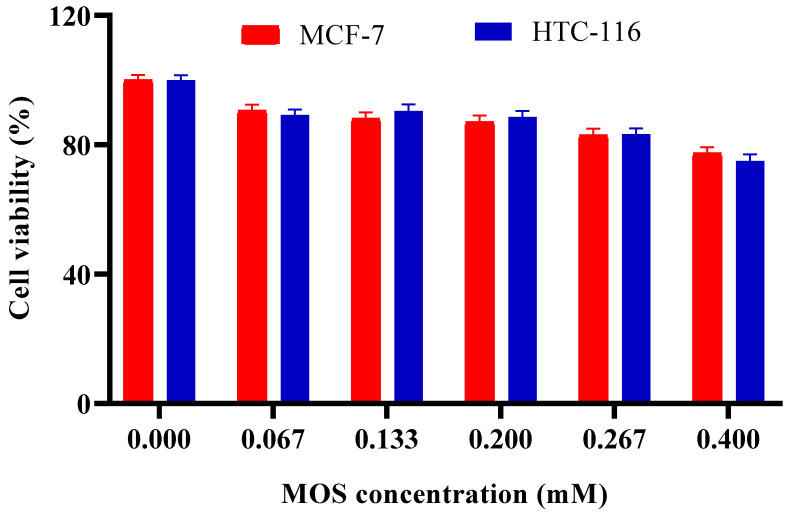
Effect of MOS concentration on viability of MCF-7 and HCT-116 cell lines. Other specifications may be found in the Methods section. All experiments have been repeated in triplicate.

Various concentrations of LBG hydrolyzates ranging from 0 to 0.4 mM were used to treat MCF-7 and HCT-116 cell lines. As shown in Figure 7, the cell viability decreased when the amount of MOSs increased. For the 0.133 mM MOS concentration, cell growth inhibition was approximately 12% and 10% for MCF-7 and HCT-116 cell lines, respectively. When 0.4 mM MOS was used, the highest inhibition was observed as 24% and 25% for MCF-7 and HCT-116 cell lines, respectively. Ghosh et al. [53] reported that copra meal-derived MOSs at a concentration of 500 μg/mL reduced the survival of HT29 cells by 50%. Similarly, guar gum hydrolysate demonstrated anti-cancer properties, decreasing the viability of Caco-2 cells by 30.95% at the same concentration. Konjac gum oligosaccharides, primarily composed of M4, induced a 30% cell death at a concentration of 1000 μg/mL. Additionally, konjac glucomannan inhibited Hep G2 cells by modulating the Bcl-2/Bax protein pathway, resulting in an altered Bax/Bcl-2 ratio [73].

## 3. Materials and Methods

### 3.1. Materials

Endo-1,4-β-Mannanase from *Aspergillus niger* was obtained from Megazyme (Bray, Ireland). Polyvinyl alcohol (PVA, Mw 89,000–98,000), locust bean gum (LBG), and 3,5-dinitro salicylic acid (DNS) were purchased from Sigma Aldrich (St. Louis, MO, USA). Genipin was obtained from BLDpharm (Songjiang, China). All reagents were of analytical grade and used without further purification.

### 3.2. Mannanase Assay

To evaluate mannanase activity, LBG was used as substrate. The main backbone of LBG is composed of β-(1→4)-linked D-mannose units, and its side chains include single-unit α-(1→6)-linked D-galactose branches. Briefly, 50 µL of the enzyme solution was combined with 250 µL of a 0.25% (*w*/*v*) LBG solution prepared in 100 mM sodium acetate at pH 5.0 and incubated at 40 °C for 15 min [68]. Following this incubation, 300 µL of DNS was added, and the mixture was boiled for 10 min. After cooling to room temperature, the solution was diluted to a final volume of 4.0 mL with distilled water, and the absorbance was measured at 540 nm. A control was prepared using the same procedure but substituting the reaction solution with an equivalent volume of LBG solution. To measure the activity of immobilized enzymes, approximately 7 mg of the encapsulated enzyme was added to the reaction tube. Mannose equivalents were determined using a standard curve for reducing sugar concentration. One unit (IU) of mannanase activity was defined as μmol of released mannose per minute under the specified assay conditions.

### 3.3. Immobilization of Mannanase

The immobilization of mannanase was performed through entrapment in PVA gel, following the protocol described by Toprak et al. [74]. A 10% PVA solution was prepared in a glass bottle and then heated in a water bath at 90 °C. After cooling this solution to room temperature, 1 mL of mannanase (0.25 mg protein/mL in 100 mM sodium phosphate at pH 7.0) was added to 2 mL of the PVA solution at 25 °C. Subsequently, samples of 100 µL of the resulting mixture were deposited onto a rigid polystyrene surface for developing gelation. The solid gel particles (PVA@mannanase) were washed with 0.1 M sodium phosphate at pH 7.0 and then stored overnight at 5 °C until use. In some instances, 1 g/L (*w*/*v*) genipin solid was dissolved in the enzyme solution prior to combining it with the PVA solution (PVA@mannanase-Gen), thereby repeating the same procedure as before.

The immobilization yield and expressed activity were calculated as described by Boudrant et al. [75].

### 3.4. Fourier Transform Infrared Spectroscopy and Scanning Electron Microscopy Analysis

Fourier transform infrared spectroscopy (FTIR) analysis was performed using the Attenuated Total Reflectance (ATR) technique on PVA (control), PVA@mannanase, and PVA@mannanase-Gen. Spectra were recorded in the range of 500–4000 cm^−1^ using a Perkin Elmer Spectrum 100 FT-IR spectrometer. The instrument was equipped with a ZnSe/diamond sampling module and a single reflection detector, operating at room temperature. A total of 32 scans were collected for each spectrum at a resolution of 4 cm^−1^.

The surface morphology of PVA (control), PVA@mannanase, and PVA@mannanase-Gen was examined using scanning electron microscopy (SEM, Quanta 650 Field Emission SEM). To reduce charging effects, the samples were coated with a thin gold layer. The SEM was operated at 15 kV, and images were captured at various magnifications ranging from 5000× to 50,000×.

### 3.5. Effects of pH and Temperature on the Activity of the Different Enzyme Preparations

The effect of pH on free and immobilized mannanase activity preparations was assessed across a range of pH values from 3.0 to 6.0, using 100 mM sodium acetate at a constant temperature of 40 °C. Moreover, the influence of the temperature on enzyme activity was examined in the temperature range of 30–60 °C at pH 5.0, under standard assay conditions. The maximum activity observed within these specific pH and temperature ranges was designated as 100%, and the activities at all other corresponding pH values and temperatures were expressed relative to this baseline activity.

### 3.6. Enzyme Kinetic Parameters

The kinetic parameters of both free and immobilized mannanase samples were assessed by testing various substrate concentrations of LBG, ranging from 0.1 to 5 mg/mL, at optimal pH and temperature conditions. The apparent Michaelis–Menten constants (*K*_m_) and maximum activity (*V*_max_) were determined from the resulting Michaelis–Menten plot.

### 3.7. Thermal Stability

The thermal stability of free and immobilized mannanase samples was assessed by incubating them at 40 °C and 55 °C in 100 mM sodium acetate at their optimum pH. At specified time intervals, aliquots of the inactivation mixtures were taken to measure the enzyme activity under optimal conditions. The residual activity was calculated as the percentage of activity at each inactivation time point relative to the initial enzyme activity, allowing for a comparison of thermal stability between the free and immobilized forms of mannanase.

### 3.8. Reusability of Immobilized Mannanase Samples

The reusability of immobilized mannanase samples was evaluated in a batch-type reactor. Ten milligrams of immobilized mannanase were incubated in 1 mL of 100 mM sodium acetate (pH 5.0) under optimal activity conditions. The reaction was initiated by adding 400 µL of LBG at a concentration of 1 mg/mL. After 15 min of the reaction, the immobilized mannanase samples were removed from the reaction medium, and the resulting product solution was analyzed at 540 nm using an activity assay. To prepare the samples for subsequent cycles, the immobilized mannanase was washed with distilled water.

### 3.9. Time-Course Hydrolysis of LBG and Analysis of the Reaction Products

Five milligrams of PVA@mannanase-Gen were mixed with 1 mL of 0.25% (*w*/*v*) LBG. The reaction was performed in 100 mM sodium acetate at pH 5.0 and 55 °C under agitation of 100 rpm for different times (1–8 h). The amount of reducing sugar was determined using DNSA method.

The hydrolysate after 8 h of reaction time was separated from the biocatalysts through filtration using 0.45 μm nylon filters to ensure the removal of all biocatalyst residues. Samples of 20 μL of filtrate were taken out and analyzed by high-performance liquid chromatography coupled with a refractive index detector (HPLC-RID). The separation of reaction products was achieved on a Supelcosil LC-NH2 (5 μm particle size, L × I.D. 25 cm × 4.6 mm) column at 40 °C. A mixture of acetonitrile/water (75/25, *v*/*v*) was used as the mobile phase at a flow rate of 0.5 mL/min. All experiments have been repeated in triplicate.

### 3.10. Breast and Colorectal Carcinoma Cell Cultures

MCF-7 cells were cultured in a DMEM culture medium with 10% FBS and 1% penicillin/streptomycin antibiotic in a 25 cm^2^ cell culture flask in an incubator at 37 °C in an atmosphere containing 95% humidity and 5% CO_2_. Moreover, HTC-116 cells were cultured in a mixture of DMEM and F12 in equal ratio, supplemented with 5% heat-inactivated FBS, 1% L-glutamine, and 1% penicillin-streptomycin antibiotic in a 25 cm^2^ cell culture flask in an incubator at 37 °C in an atmosphere containing 95% humidity and 5% CO_2_. When the cells achieved an 80% uptake, they were trypsinized, resuspended, and transferred to a 75 cm^2^ cell culture flask. Cell counting was performed with trypan blue [76]. Phase-contrast microscopy (Thermo Fisher Scientific, Evos XL Core, Oxford, UK) was used to evaluate the appearance of the cells. At the 80% confluency, the cells were trypsinized, resuspended, and counted to assess cell density. Cells were inspected under a phase-contrast microscope to evaluate their appearance.

### 3.11. Assessment of Cell Survival by CCK-8 Method

MCF-7 and/or HCT-116 cells were seeded into 96-well plates containing 200 μL of DMEM and cultured for about 24 h in an incubator at 37 °C in an atmosphere containing 95% humidity and 5% CO_2_ [77]. The hydrolysate obtained from PVA@mannanase-Gen-catalyzed hydrolysis of LBG after 6 h of reaction time was separated from the biocatalysts through filtration using 0.45 μm nylon filters and used as MOS source. A 100 µL volume of the solution was added to the wells at different MOS concentrations (0.0–0.4 mM) and was placed in the incubator under the same conditions. At the end of 24 h, 100 µL of the medium was taken and transferred to a new plate. Then, 100 µL of fresh medium and 20 µL of CCK-8 solution were added and kept in the incubator for 4 h. Optical density (OD) at 450 nm was obtained with a microplate reader (BioTek, Epoch2 microplate Reader, MA, USA). The culture medium and CCK-8 were used as a blank, and untreated MCF-7 or HCT-116 cells were used as a control. Cell viability was then calculated by comparing the absorbance of treated samples to that of control (untreated) samples, typically using the following formula:

$$Cell\;viability\;\left(\%\right)\;=\;\frac{{(A}_{t}\;-\;A_{b})}{{(A}_{c}\;-\;A_{b})}\;\times \;100$$

where *A_t_*, *A_c_*, and *A_b_* are the absorbances of the treated sample, control sample, and blank sample at 450 nm, respectively.

### 3.12. Statistical Analysis

All experiments were performed in triplicate. Statistical analysis was conducted using analysis of variance (ANOVA) to determine significant differences among experimental groups. Group means were compared using the Least Significant Difference (LSD) method in SigmaPlot software (Version 12), with a significance level set at *p* < 0.05.

## 4. Conclusions

Mannanase from *A. niger* was immobilized in PVA, and the obtained immobilized mannanase was further treated with genipin. The enzyme optimal pH did not change after immobilization, whereas the immobilization of mannanase resulted in an increase in the optimal temperature from 40 °C to 55 °C at pH 5.0. PVA@mannanase-Gen exhibited a 2.3-fold higher catalytic efficiency compared to the free enzyme. After five reuses, at least 75% of the initial activity remained for the immobilized mannanase preparations. The MOS produced under these conditions showed moderate antiproliferative effects against MCF-7 and HCT-116 cells after 24 h; however, these preliminary in vitro observations require further mechanistic studies and in vivo validation before any therapeutic potential can be established. In conclusion, the immobilization of mannanase in PVA and subsequent treatment with genipin enhanced its thermal stability, catalytic efficiency, and reusability. However, although PVA–genipin systems offer a good biocompatibility and enzyme stabilization, they also present limitations. PVA’s high hydrophilicity can cause excessive water retention and swelling, reducing the mechanical strength and potentially affecting enzyme retention. Genipin, while safer than other crosslinker agents, displays slow reaction kinetics, which may lead to long biocatalyst preparation times and less uniform network formation. To overcome these drawbacks, alternative strategies include blending PVA with polymers with a lower tendency to swell (e.g., chitosan, alginate, or cellulose derivatives) and incorporating inorganic fillers to enhance rigidity. If the biocompatibility and low toxicity are not strict requirements (e.g., in an energy production process), faster or dual-crosslinking systems may be used.

## Figures and Tables

**Table 1:** Immobilization yields and activity of recovery values of PVA@mannanase and PVA@mannanase-Gen for different protein loadings. Other specifications can be found in the Methods section.

Loaded Protein Concentration (mg/mL)	Immobilization Yield (%)	Expressed Activity (mannanase-Gen) (%)	Expressed Activity (PVA@mannanase) (%)	Expressed Activity (PVA@mannanase-Gen) (%)
0.125	100	125.2	34.8	45.2
0.25	100	122.3	86.6	119.2
0.5	100	110.1	23.2	28.6

**Table 2:** The apparent kinetic parameters of free mannanase, PVA@mannanase, and PVA@mannanase-Gen for LBG at optimal pH and temperature of each preparation.

Biocatalyst	*K*_m_ (mg mL^−1^)	*V*_max_ (U/mg Protein)	Catalytic Efficiency Ratio	Reference
MC	8.1	135	0.50	[25]
MB-C	6.7	67.1	0.37	[25]
Free 1,4-β-mannanase	10.7	106		[27]
Man/Cel5B	4.8	2.56	0.05	[27]
Free 1,4-β-mannanase	8.44	55.36		[67]
Immobilized on glutaraldehyde-activated chitosan nanoparticles	7.74	12.10	0.24	[67]
Free 1,4-β-mannanase	25	2500		[28]
NaAlg-β-CD-β-mannanase	36	2252.25	0.63	[28]
Free 1,4-β-mannanase	10.2	3.24		[68]
Man-CaAlg	19.4	6.17	1.0	[68]
Free mannanase	25.8 ± 0.04	55.0 ± 0.07	-	This study
PVA@mannanase	69.6 ± 0.05	34.4 ± 0.08	0.24	This study
PVA@mannanase-Gen	14.0 ± 0.03	68.9 ± 0.06	2.30	This study

## Data Availability

The data presented in this study are available on request from the corresponding author.

## Abbreviations

The following abbreviations are used in this manuscript:

APTES	(3-Aminopropyl)triethoxysilane
FTIR	Fourier transform infrared spectroscopy
HTC-116	Human colon cancer cell
k_cat_	Turnover number
K_m_	Michaelis–Menten constant
MCF-7	Human breast cancer cell
MOS	Mannooligosaccharide
PEI	Polyethyleneimine
PVA	Polyvinyl alcohol
PVA@mannanase	Immobilized mannanase in polyvinyl alcohol hydrogel
PVA@mannanase-Gen	Immobilized mannanase modified with genipin in polyvinyl alcohol hydrogel
SEM	Scanning electron microscopy
V_max_	Maximum velocity
